# Thyrotoxicosis as a rare presentation in acute suppurative thyroiditis: a case report

**DOI:** 10.1186/s13256-023-04119-1

**Published:** 2023-10-14

**Authors:** Zeynab Seyedjavadeyn, Seyed Amir Miratashi Yazdi, Alireza Samimiat, Matin Vahedi

**Affiliations:** 1 Department of Surgery, Sina Hospital, Tehran University of Medical Sciences, Tehran, Iran; 2 Department of General Thoracic Surgery, Sina Hospital, Tehran University of Medical Sciences, Tehran, Iran

**Keywords:** Thyroiditis, Suppurative, Surgery, Thyrotoxicosis

## Abstract

**Background:**

Acute suppurative thyroiditis is a rare and potentially life-threatening disease. A few cases of acute suppurative thyroiditis associated with thyrotoxicosis have been reported in adults. We report a case of acute suppurative thyroiditis that was associated with thyrotoxicosis.

**Case presentation:**

We report the case of a 49-year-old Iranian female presented with a painful neck swelling for a week. Computed tomography showed a well-defined gas-filled collection in the left thyroid lobe with an enhancing margin. The patient underwent two-phase surgery, first left thyroid abscess drainage and then total thyroidectomy. The result of histopathology examination was multinodular goiter with abscess formation without malignancy.

**Conclusion:**

Abscess formation and thyrotoxicosis is a very rare condition that occurs at the same time in acute suppurative thyroiditis. Despite antibiotic therapy being the first line of treatment, surgery is also required when antibiotic therapy fails.

## Introduction

Acute suppurative thyroiditis (AST) is an uncommon condition that occurs in 0.1–0.7% of thyroid diseases [1]. It is an infection in the thyroid gland that progresses to an abscess. This is a serious, rare, and potentially fatal condition [1].

Approximately 92% of those affected are under the age of 49 years, with a slight female predominance [5, 6, 16]. Progression to abscess formation is even rarer since the thyroid gland’s structural and physiological features protect it against infection. Owing to the complete encasement of the gland with separation from other organs, its rich blood supply, high iodine content, and good lymphatic drainage, the thyroid is relatively resistant to most infections [2, 4]. Although bacteria are the most common causative microorganisms, fungi have been implicated in immunocompromised patients [5]. Immunosuppression and pyriform sinus fistulae are known as the most common contributing factor for AST [16]. It has been reported that 5–10% of AST cases show abnormal levels of thyroid hormone, but cases accompanied by thyrotoxicosis are quite rare. Thyrotoxicosis can be due to damage or destruction of thyroid tissue by infectious agents, leading to the release of stored thyroid hormone into the bloodstream. The diagnosis is typically made through blood tests that measure levels of thyroid-stimulating hormone (TSH) and thyroid hormones (T3 and T4) and clinical signs include weight loss, increased appetite, sweating, anxiety, tremors, and palpitations [3, 16].

Here, we report a case of AST accompanied with thyrotoxicosis in a 49-year-old woman who had no special medical history other than type 2 diabetes mellitus and had no abnormal anatomical structure.

## Case report

**Fig. 1:**
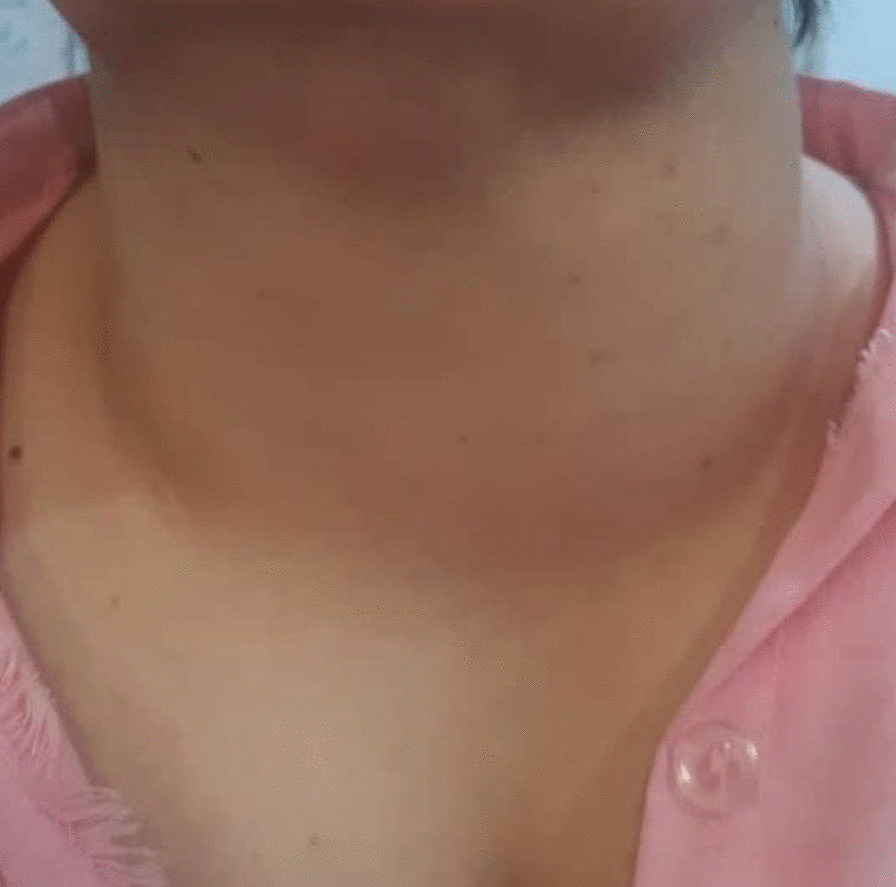
Neck swelling, predominantly on the left side, and difficulty breathing

A 49-year-old Iranian woman presented with 1 week of gradually painful neck swelling, predominantly on the left side, and difficulty breathing (Fig. 1). Her symptoms started 1 month previously; she had developed sore throat, neck pain, and tachycardia, and she was diagnosed with thyroiditis (C-reactive protein increased to 75 mg/dL, TSH: 0.005 (0.4–6.2 Miu/ml), T4: 18.6 (4.87–11.72 micg/dl)) which was treated by an endocrinologist with prednisolone and propranolol. But since 1 week ago, the left side of the neck had been gradually swelling.

Her past medical history was remarkable for type 2 diabetes controlled with metformin. Family and surgical history were negative. She had no history of neck trauma. The patient was alert but showed signs of acute illness. Her blood pressure measured 120/80 mmHg, pulse rate was 105 bpm, respiratory rate was 22 times per minute, oxygen saturation was 94% on room air, and the sublingual temperature was 38.5 °C. On the basis of examinations of her head and neck, a lump was detected and palpitation of the lump was accompanied by minor tenderness. The lump was fixated and firm. There was no finding of tonsillar hypertrophy and enlargements of lymph nodes.

The blood assay revealed that white blood cell (WBC) count was 20,900/mm^3^ (neutrophils, 88.9%). The erythrocyte sedimentation rate (ESR) increased to 91 mm/hour, and C-reactive protein increased to 126 mg/dL. The endocrinology report showed that T3 was 1.0 ng/dL (normal range, 0.52–1.85 ng/dL), free T4 was 9.5 micg/dl (normal range, 4.87–11.72 micg/dl), and thyroid stimulating hormone (TSH) was 0.01 μIU/mL (normal range, 0.4–6.2 μIU/mL).

**Fig. 2:**
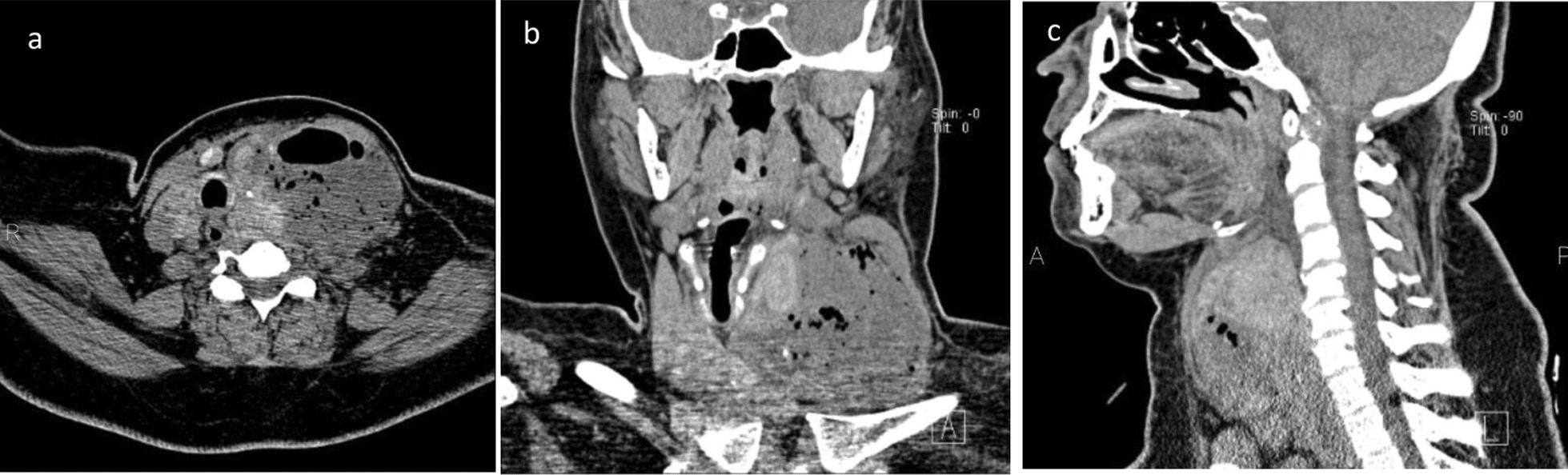
Axial, coronal, and sagittal (**a**, **b**, **c**) computed tomography images showing a well-defined gas-filled collection with enhancing margins in the left thyroid lobe plus widespread edema surrounding the left thyroid lobe; the trachea was shifted to the right side of the neck

On ultrasound examination, left thyroid lobe was enlarged and showed a complicated thick-walled cyst with air-echogenic foci. It pushed left common carotid artery. Right thyroid lobe was 45 × 21 × 19 mm in size with multiple isoechoic nodules measuring < 20 mm. CT scan of the neck and upper chest revealed a well-defined gas-filled collection with enhancing margins in the left thyroid lobe, and there was widespread edema surrounding the left thyroid lobe; the trachea was shifted to the right side of the neck, and a number of reactive lymph nodes were discovered (Fig. 2).

IV antibiotic (imipenem) was initiated. Owinbg to difficult breathing, the thyroid abscess was drained by a longitudinal cervical incision at the site of maximum bulging. Abscess secretions were sent for culture and smear, and *Escherichia coli* was grown. Owing to its sensitivity to imipenem, the antibiotic was not changed. After surgery, her symptoms were much improved, WBC decreased, ESR was 42, and CRP 26. On the 7th postoperative day, despite antibiotic treatment, WBC and acute-phase reactant increased. US revealed a small abscess in left thyroid lobe. So, by collar incision, the neck was explored which extended to last incision. Because of multi abscess in left lobe and multi nodules in bilateral lobe, we performed total thyroidectomy. The pathological findings revealed multinodular goiter with inflammation accompanied by necrotic tissue and abscess and foamy macrophage aggregation and focal calcification noted in left lobe.

**Fig. 3:**
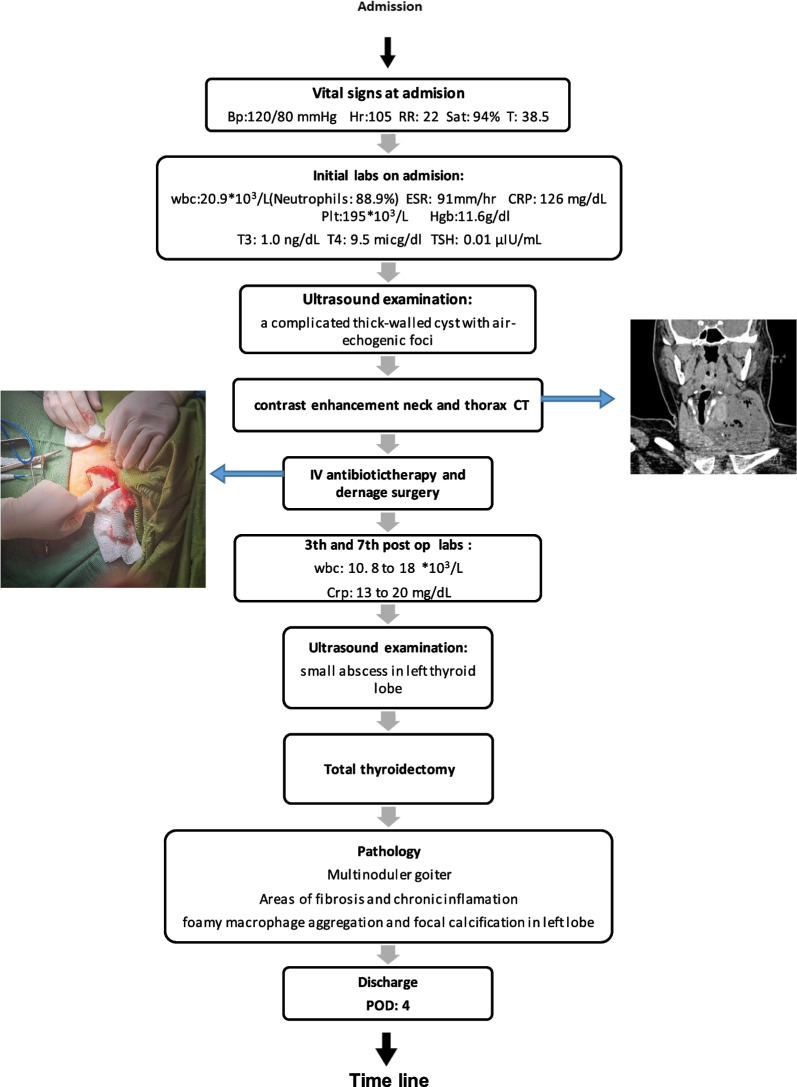
Timeline from admission to discharge

After the surgery, the patient showed complete recovery and was discharged from the hospital 4 days after surgery (Fig. 3).

## Discussion

Thyroiditis caused by an infectious agent might be acute or chronic. AST is a rare life-threatening endocrine emergency of the thyroid gland. Approximately 92% of those affected are under the age of 49 years, with a slight female predominance [6, 16]. In this case, age was older than the usual age which was 49 years.

The majority of individuals with acute suppurative thyroiditis fall into one of four categories: (1) thyroiditis caused by bacterial infection through a pyriform sinus fistula, (2) infection of a thyroid nodule or cancer, (3) infection caused by esophageal rupture owing to foreign-body ingestion or esophageal malignancy, and (4) infection of the normal thyroid gland [7, 16]. Hematogenous spread, direct invasion of neighboring tissues, lymphatic spread, direct trauma, and persistent thyroglossal duct are all possible routes of infection [8]. Up to 95% of AST develops in the left thyroid lobe. Thyroid abscess in children is most often caused by anatomical abnormality of the hypopharyngeal area, which leads to the formation of a pyriform sinus fistula [9]. In adults, proposed routes of infection for AST include lymphatic or hematogenous spread, direct inoculation of the thyroid or surrounding anatomy, direct extension of an abscess, and spread through a pyriform sinus fistula, usually in the setting of preexisting thyroid disease or an immunocompromised patient. Abnormal thyroid structures, such as multinodular goiter, nodules, or malignancies, have been postulated to make the thyroid gland more susceptible to a suppuration state [2, 11]. In this case, the patient had past medical history of diabetes mellitus and surgical pathology showed multi nodular goiter.

Early diagnosis of AST is critical to avoid a catastrophic effect. The laboratory findings include increased erythrocyte sedimentation rate (ESR) and leukocyte count [10, 16]. When it comes to thyroid function, although the majority of individuals are euthyroid and no thyroid autoantibodies are detected, both thyrotoxicosis (12%) or hypothyroidism (17%) may also be present [11, 12]. Transient thyrotoxicosis can be caused by diffuse thyroid inflammation, and follicular disruption leads to the release of preformed thyroid hormones into the bloodstream [5]. AST accompanied by thyrotoxicosis is difficult to differentiate from subacute thyroiditis and thyroid cancer, resulting in cases with delayed diagnosis due to misdiagnosis as subacute thyroiditis [13]. In the current case, she had thyrotoxicosis.

The ideal treatment strategy for acute suppurative thyroiditis is still being discussed [7]. Supportive treatment and antibiotic therapy are used to treat AST in the beginning. For severe infections, parenteral antibiotics are necessary, and the choice of antibiotic is guided by microscopic examination, staining, and culture of the FNAC [14]. If antibiotic therapy fails, surgery, such as incision drainage, may be required. Drainage has been reported to be effective in many reports, and it can be repeated if the abscess remains or if there is progression [1]. In unstable individuals with compromised airways, drainage is critical. In extreme situations, in patients who do not respond to appropriate antibiotic therapy and drainage, open surgery with complete, near-total, or hemithyroidectomy can be used to relieve pressure symptoms [15]. In our case, the patient underwent abscess drainage first, but owing to the lack of response to antibiotic treatment and multinodular goiter in both thyroid lobes, the patient underwent total thyroidectomy surgery.

## Conclusion

AST progressing to abscess formation is a rare but potentially life-threatening condition that may be easily misdiagnosed as a result of its low incidence. Thyrotoxicosis also can be an uncommon clinical feature of AST that could be due to thyroid inflammation. After implementing treatment with antibiotics and surgical intervention, there was good postoperative prognosis.

## Data Availability

The datasets used and/or analyzed during the present study are available from the corresponding author upon reasonable request.

## Abbreviations

AST: Acute suppurative thyroiditis
ESR: Erythrocyte sedimentation rate
WBC: White blood cells
TSH: Thyroid stimulating hormone
FNAC: Fine needle aspiration cytology
